# Stably Expressed Housekeeping Genes across Developmental Stages in the Two-Spotted Spider Mite, *Tetranychus urticae*

**DOI:** 10.1371/journal.pone.0120833

**Published:** 2015-03-30

**Authors:** Chunxiao Yang, Huipeng Pan, Yong Liu, Xuguo Zhou

**Affiliations:** 1 Hunan Academy of Agricultural Sciences, Institute of Plant Protection, Changsha, Hunan, China; 2 Department of Entomology, University of Kentucky, Lexington, Kentucky, United States of America; Nazarbayev University, KAZAKHSTAN

## Abstract

Quantitative real-time PCR (qRT-PCR) is a reliable and reproducible technique for measuring mRNA expression. To facilitate gene expression studies and obtain more accurate qRT-PCR analysis, normalization relative to stable housekeeping genes is mandatory. In this study, ten housekeeping genes, including *beta-actin (Actin) *, *elongation factor 1 α (EF1A) *, *glyceralde hyde-3-phosphate dehydrogenase (GAPDH) *, *ribosomal protein L13 (RPL13) *, *ribosomal protein 49 (RP49) *, *α-tubulin (Tubulin) *, *vacuolar-type H+-ATPase (v-ATPase) *, *succinate dehydrogenase subunit A (SDHA) *, *28S ribosomal RNA (28S) *, and *18S ribosomal RNA (18S) * from the two-spotted spider mite, *Tetranychus urticae*, were selected as the candidate reference genes. Four algorithms, *geNorm*, *Normfinder*, *BestKeeper*, and the *ΔC_t_* method, were used to evaluate the performance of these candidates as endogenous controls across different developmental stages. In addition, *RefFinder*, which integrates the above-mentioned software tools, provided the overall ranking of the stability/suitability of these candidate reference genes. Among them, *PRL13* and *v-ATPase* were the two most stable housekeeping genes across different developmental stages. This work is the first step toward establishing a standardized qRT-PCR analysis in *T*. *urticae* following the MIQE guideline. With the recent release of the *T*. *urticae* genome, results from this study provide a critical piece for the subsequent genomics and functional genomics research in this emerging model system.

## Introduction

Quantitative real-time PCR (qRT-PCR) is a rapid and reliable method for the detection and quantification of gene expression during different biological processes [1]. Although qRT-PCR analysis has been a primary tool in the molecular biology research, limitations still exist, including variation in RNA extraction, reverse transcription and normalization, and PCR efficiency [2, 3]. To ensure the accuracy, a critical component in qRT-PCR analysis is to normalize data by measuring in parallel the expression of a reference gene from the same samples. Housekeeping genes, involved in basic cellular functions, are typically maintaining stable and constitutive expression in all cells and regardless of physiological conditions. Consequently, housekeeping genes have been widely adopted in many molecular and genomics studies as a reference for calibration purposes, including qRT-PCR analysis [1,4].

The two-spotted spider mite, *Tetranychus urticae* Koch, is one of the most important pest species worldwide. Considered as the most polyphagous species within the family of Tetranychidae, *T*. *urticae* can infest nearly 800 plant species, including peppers, tomatoes, potatoes, beans, maize, strawberries, and ornamental plants such as roses [5]. *T*. *urticae* lays its egg on the leaf, damages host plants by sucking cell contents from the leaf, and leaves tiny pale spots or scars where the green epidermal cells have been destroyed [5]. Although the individual lesions are small in size, attacks by hundreds and thousands of spider mites can significantly reduce the photosynthetic capability of plants [5]. The control of *T*. *urticae* has traditionally relied on synthetic insecticides and acaricides. As a result, *T*. *urticae* has developed resistance to almost every major active compound [6]. With the advent of *T*. *urticae* genome [7], the first complete genome being sequenced from a chelicerate species, there is an unprecedented opportunity to investigate the genetic basis of pesticide resistance and other biological phenomena in *T*. *urticae* [6, 8, 9]. To take advantage of these genomic resources, establishing a standardized qRT-PCR procedure in *T*. *urticae* following the MIQE (Minimum Information for publication of Quantitative real-time PCR Experiments) guidelines [10] will be instrumental for the subsequent genomics and functional genomics studies in this emerging pest model.

The objective of this study was to address an important aspect of gene expression studies in *T*. *urticae*: the selection and validation of appropriate reference genes with stable expression under different developmental stages. Here, ten housekeeping genes, including *beta-actin* (*Actin*), *elongation factor 1 α* (*EF1A*), *glyceralde hyde-3-phosphate dehydrogenase* (*GAPDH*), *ribosomal protein L13* (*RPL13*), *ribosomal protein 49* (*RP49*), *α-tubulin* (*Tubulin*), *vacuolar-type H+-ATPase* (*v-ATPase*), *succinate dehydrogenase subunit A* (*SDHA*), *28S ribosomal RNA* (*28S*), and *18S ribosomal RNA* (*18S*) from the *T*. *urticae* genome were selected as the candidate reference genes [7]. The stability of these reference genes was investigated across different developmental stages. To validate the recommendations, the effectiveness of these candidates was further examined by qRT-PCR analysis using *CYP392D8*, a P450 gene associated with the abamectin resistance [9].

## Materials and Methods

### Ethics Statement

The *Tetranychus urticae* colony was collected from honeyvine milkweed, *Cynanchum laeve* (*syn*. *Ampelamus albidus*) in a greenhouse (20–28°C) at the University of Kentucky. No specific permit was required for the described collections. *T*. *urticae* is a common mite species with agricultural importance in the USA.

### Sample preparation

The *T*. *urticae* was maintained in a climate chamber at 23°C with a photoperiod of 14: 10 (L: D) and 50% relative humidity. Fifteen adult females were allowed to oviposit for 12 h on a milkweed leaf resting on wet filter paper in a petri dish (9 cm diameter). When the adults were removed, there were approximately 100 eggs in each petri dish. One week after the egg eclosion, approximately 60 nymphs (a mix of protonymphs and deutonymphs) were collected. Adults were collected directly from the milkweed, and a total of 40 mixed-sex individuals were used in one biological replicate. All samples were preserved in 1.5 ml microcentrifuge tubes and stored at −80°C after being snap frozen in liquid nitrogen. For egg, nymph, and adult samples, each sample had three technical replications, and each developmental stage was independently collected three times.

### Total RNA extraction and cDNA synthesis

*Tetranychus urticae* samples (100 eggs, 60 nymphs, or 40 adults) were homogenized in a 1.5 ml RNase-free microcentrifuge tube containing 400 μl TRIzol reagent (Invitrogen, Carlsbad, CA). The whole body homogenates were centrifuged at 12000 *g* for 15 min and then supernatant was transferred to a new 1.5 ml microcentrifuge tube. A volume of 100 μl chloroform was added to the supernatant and then the mixture was incubated at room temperature for 10 min and then centrifuged at 4°C, 12000 *g* for 15 min. After that, the supernatant was transferred to a new 1.5 ml microcentrifuge tube, 200 μl Isopropyl alcohol was added to it, and the mixture precipitated at room temperature for 10 min. Then, the supernatant was discarded after the mixture was centrifuged at 4°C, 12000 *g* for 8 min, and then 500 μl 75% alcohol was added and centrifuged at 4°C, 7500 *g* for 5 min to wash the pellet. Finally, the pellet was air dried for 5 min and then dissolved in 30 μl ddH_2_O. DNAse treated total RNA was denatured at 75°C for 5 min and immediately chilled on ice. The concentration of RNA from the egg, nymph, and adult was quantified with a NanoDrop 2000c spectrophotometer with the result for egg (83.2 ± 17.2 ng/μl), nymph (104.0 ± 10.3 ng/μl), and adult (113.7 ± 23.1 ng/μl). First strand cDNA was synthesized from 0.1 μg of total RNA with M-MLV reverse transcription kit (Invitrogen, USA) using a random N primer according the manufacturer’s recommendations. The cDNA was diluted five-fold for the subsequent qPCR studies.

### Reference gene selection and primer design

Table 1 lists the candidate reference genes and primer sets associated with these candidates. PCR amplifications were performed in 50 μl reactions, containing 10 μl 5×PCR Buffer (Mg^2+^ Plus), 1 μl dNTP mix (10 mM of each nucleotide), 5 μl of each primer (10μM each), and 0.25 μl of Go Taq (5 u/μl) (Promega). The PCR parameters were as follows: one cycle of 94°C for 3 min; 35 cycles of 94°C for 30 s, 59°C for 45 s and 72°C for 1 min; and a final cycle of 72°C for 10 min. Amplicons of the expected size were purified and cloned into the pCR4-TOPO vector (Invitrogen, Carlsbad, CA) for the sequencing confirmation.

**Table 1:** Primer set for qRT-PCR analysis.

Gene	Accession No.	Primer sequences(5’-3’)	Length(bp)	E(%)*	R^2,^ **
*EF1A*	GU198154	F:AGGGAGCTAAATTGGAAGGTAAA	93	96.9	0.9968
	(tetur02g11060) ^†^	R: GTGGAAGTCGAAGTGCCTTAT			
*SDHA*	JN881329	F: TGGAGCCGGATGTTTGATTAC	100	95.7	0.9915
	(tetur08g03210)	R: GGCCACAGGTGCATATCTTT			
*GAPDH*	JN881330	F: CGATGCGCCTATGTTTGTTATG	99	93.5	0.9989
	(tetur25g00250)	R: GGAGCAAGACAGTTGGTTGTA			
*Tubulin*	JN881327	F: GCTGCCATTGCTGCTATTAAG	119	100.2	0.9968
	(tetur03g00230)	R: GCTAAATCTCCTCCAGGAACAA			
*RPL13*	JN881328	F: GCTCACAGCCTATGAAGGTATT	90	96.2	0.9976
	(tetur08g05440)	R:AGAACTTACGACCTCCTTGTAATC			
*RP49*	tetur18g03590	F: AAATTAAGAGGAACTGGCGAAA	120	101.2	0.9980
		R: GCATGTGTCTGGTGGCTTT			
*18S*	AF062961	F: CCGCCCTAGTTCTAACCATAAA	132	94.4	0.9970
	(tetur05g09306)	R: GTTTCAGCTTTGCAACCATACT			
*28S*	AY750693	F: GGATCCGTAACTTCGGGATAAG	96	93.5	0.9876
	(tetur01g03280)	R: CACCAACCAGTCTCGGTATTT			
*v-ATPase*	DQ988698	F: CCCGAAGAGATGATCCAAACTG	99	97.1	0.9963
	(tetur03g05970)	R: CGGTAAACCTGATGCTGAGAAA			
*Actin*	JN881324	F: ATCACCAACTGGGATGATATGG	127	96.8	0.9984
	(tetur03g09480)	R: GGAGCTTCTGTAAGGAGAACTG			

*: PCR efficiency (calculated from the standard curve)

**: Regression coefficient

†: http://bioinformatics.psb.ugent.be/orcae/overview/Tetur

### Quantitative real-time PCR

Gene-specific primers (Table 1) were used in qRT-PCR reactions (20 μl), containing 7 μl of ddH_2_O, 10 μl of 2×SYBR Green MasterMix (Bio-Rad), 1 μl of each specific primer (10 μM), and 1 μl of first-strand cDNA template. The qPCR program included an initial denaturation for 3 min at 95°C followed by 40 cycles of denaturation at 95°C for 10 s, annealing for 30 s at 55°C, and extension for 30 s at 72°C. For melting curve analysis, a dissociation step cycle (55°C for 10 s, and then 0.5°C for 10 s until 95°C) was added. The reactions were set up in 96-well format Microseal PCR plates (Bio-Rad) in triplicates.

Reactions were performed in a MyiQ single Color Real-Time PCR Detection System (BioRad). The presence of a single peak in the melting curve analysis was used to confirm gene-specific amplification and to rule out the non-specific amplification and the generation of primer-dimer. qRT-PCR efficiency was determined for each gene using slope analysis with a linear regression model. Relative standard curves for the transcripts were generated with serial dilutions of cDNA (1/5, 1/25, 1/125, 1/625, and 1/3125). The corresponding qRT-PCR efficiencies (E) were calculated according to the equation: E = (10^[-1/slope]^ -1)×100.

### Stability of gene expression

All biological replicates were used to calculate the average *C*_*t*_ value. The stability of the ten housekeeping genes was evaluated by algorithms *geNorm* [1], *NormFinder* [11], *BestKeeper* [12], and the comparative *ΔC*_*t*_ method [13]. Finally, we compared and ranked the tested candidates based on a web-based analysis tool *RefFinder* (http://www.leonxie.com/referencegene.php).

### Validation of reference gene selection

*CYP392D8*, a cytochrome P450 associated with abamectin resistance in *T*. *urticae* [9], was used as a target gene to evaluate the performance of candidate reference genes. *CYP392D8* expression was profiled in three life stages: egg, nymph, and adult. Two different normalization factors (NFs) were calculated based on (1) the geometric mean of the genes with the lowest *Geomean* value (as determined by *RefFinder*), and (2) a single reference gene with the lowest or highest *Geomean* value. Relative quantification of *CYP392D8* expression was calculated using the 2^-ΔΔ*Ct*^ method [14].

## Results

### Validation and transcriptional profiling of candidate reference genes

Initially, ten candidate reference genes were investigated by reverse transcription polymerase chain reaction (RT-PCR). All genes examined in *T*. *urticae* were visualized as a single amplicon with expected size on a 1.5% agarose gel (S1 Fig.). All amplicons were sequenced and displayed 100% identity with their corresponding transcripts. Furthermore, gene-specific amplification of these genes was confirmed by a single peak in real-time melting-curve analysis (S2 Fig.). A standard curve was generated for each gene, using five-fold serial dilution of the pooled cDNAs. The correlation coefficient and PCR efficiency for each standard curve are shown in Table 1.

**Fig 1:**
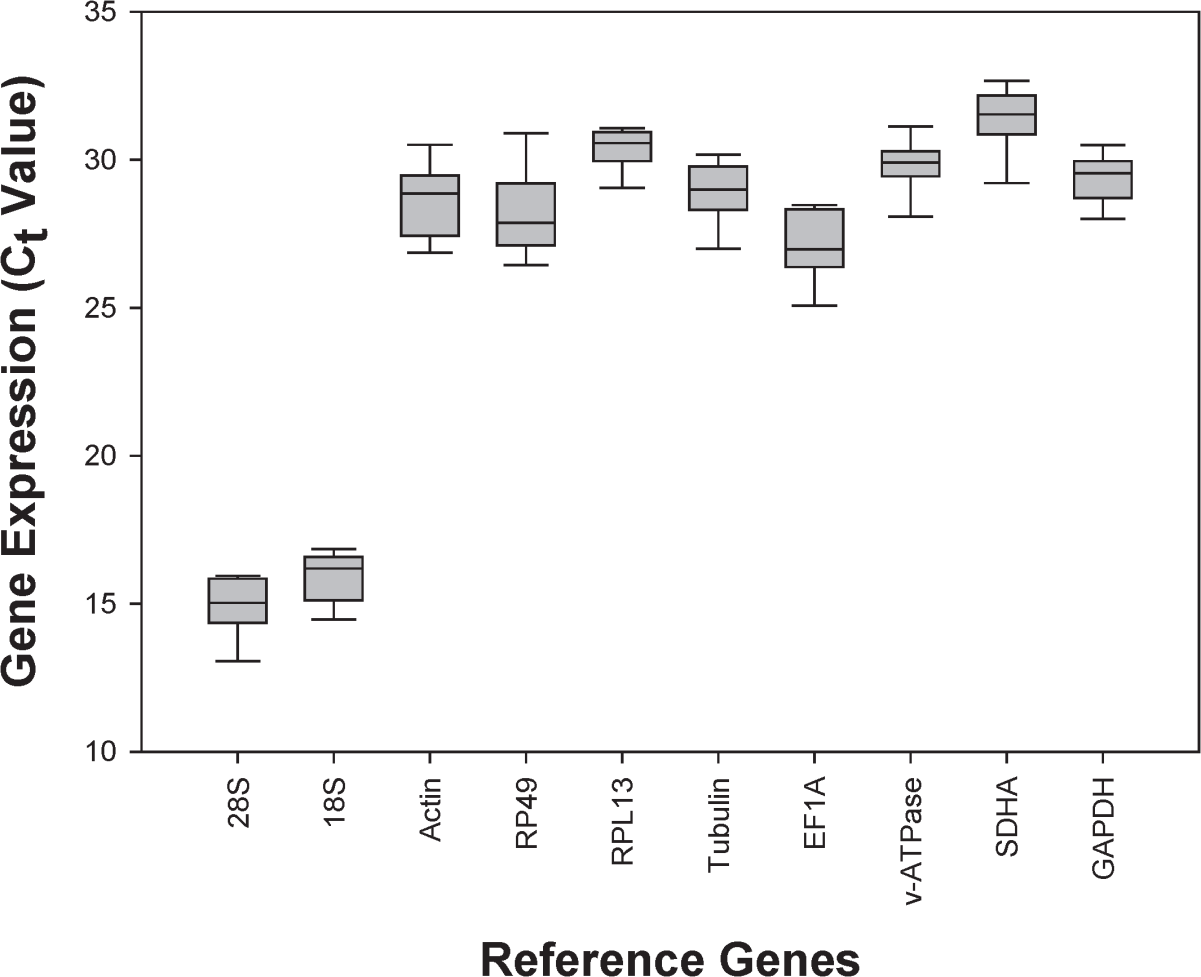
Expression profile of candidate reference genes in *Tetranychus urticae*. The expression of candidate reference genes was documented in *C*_*t*_-value. The median is represented by the line in the box. The interquartile rang is bordered by the upper and lower edges, which indicate the 75^th^ and 25^th^ percentiles, respectively.

The mean and standard deviation (SD) of the *C*_*t*_ values for egg, nymph, and adult were summarized in S1 Table. With high SD values, the expression of *RP49*, *Actin*, and *EF1A* varied substantially. In contrast, *18S*, *GAPDH*, and *RPL13* had the least variable expressions reflected in their low SD values. Additionally, *SDHA* (*C*_*tavg*_ = 31.39), and *RPL13* (*C*_*tavg*_ = 30.39) had the highest *C*_*t*_ values, suggesting that they are the least expressed gene candidates in egg, nymph, and adult. *18S* (*C*_*tavg*_ = 15.89) and *28S* (*C*_*tavg*_ = 14.94) had the lowest *C*_*t*_ values, indicating that they are the most expressed gene candidates in eggs, nymphs, and adults (Fig. 1, S1 Table).

### Optimal number of reference genes for normalization

**Fig 2:**
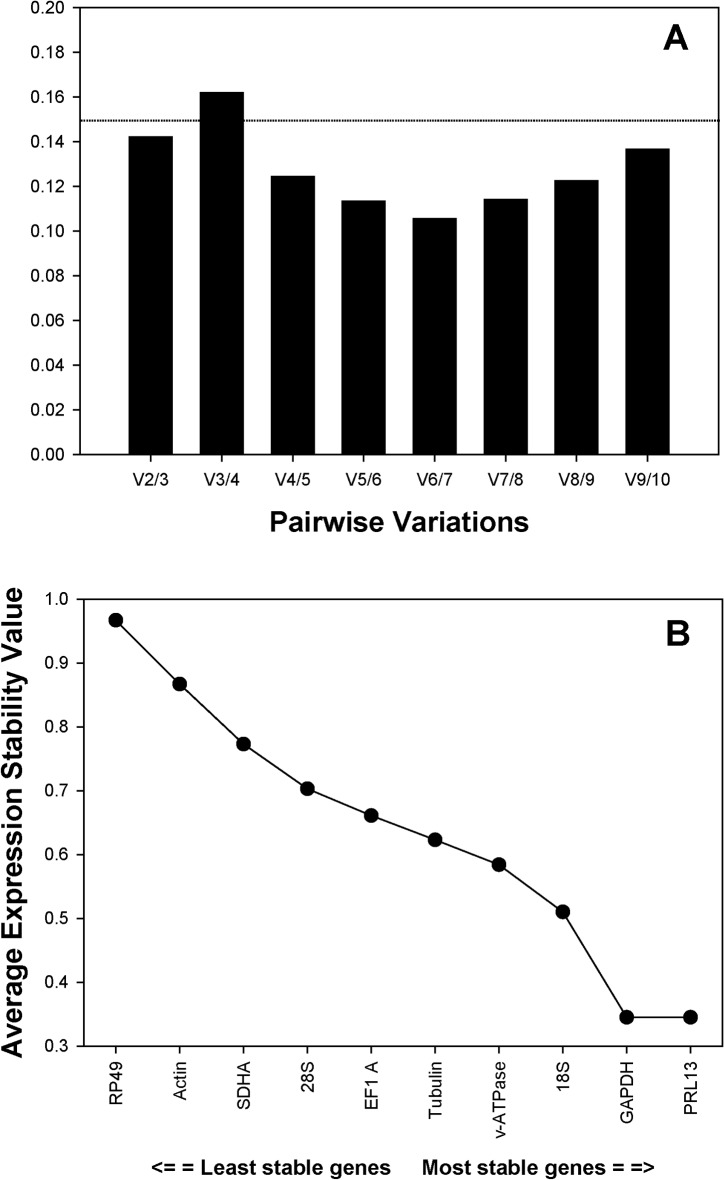
Quantitative and qualitative analyses based on *geNorm*. A) Pairwise variation (V) analysis. *geNorm* first calculates an expression stability value (M) for each gene and then compares the pair-wise variation (V) of this gene with the others. The pair-wise variation (V_n_/V_n+1_) was analyzed between the normalization factors NF_n_ and NF_n+1_ by the *geNorm* software to determine the optimal number of references genes required for normalization. A threshold of V<0.15 was suggested for the valid normalization [1]. B) Ranking of the 11 housekeeping genes based on the stability value (M).

To decide the minimal number of genes mandatory for normalization, the V-value was computed by *geNorm*. Beginning with the two most stable genes, the software automatically adds another gene following a step-wise format to recalculate the normalization factor ratio. If the newly constructed V-value is lower than the 0.15 cut-off value, the starting number of gene pairings will be sufficient for the consistent normalization. Otherwise, additional genes need to be incorporated until the V-value reach the threshold, i.e., no significant changes in the normalization factor. The first V-value<0.15 was V2/3 (Fig. 2A), suggesting that the pairing of the two most stable reference genes was reliable enough for the normalization throughout the developmental stages.

### Determining the best candidate reference genes based on the four statistical algorithms

*GeNorm* bases its ranking on the geometric mean of the SD of each transformed gene set of pair combinations (M-value). The lower the M-value is, the higher the ranking. Recommended M values for *geNorm* are M<0.5 for homogeneous samples and M<1 for heterogenous samples [15].

*RPL13* and *v-ATPase* were co-ranked as the most stable reference genes (M = 0.35). The overall ranking of candidate reference genes from the most stable to the least stable was: *RPL13*, *GAPDH*, 18S, *v-ATPase*, *Tubulin*, *EF1A*, *28S*, *SDHA*, *Actin*, and *RP49* (Fig. 2B).

*ΔC*_*t*_ method ranks the stability of candidate reference genes based on the pair-wise comparisons. Using raw *C*_*t*_ value, the average SD of each gene set is inversely proportional to its stability. As shown in S2 Table and Table 2, *RPL13* (0.76) was the most stable reference gene. The overall ranking of candidate reference genes from the most stable to the least stable was: *RPL13*, *v-ATPase*, *18S*, *Tubulin*, *GAPDH*, *28S*, *EF1A*, *SDHA*, *Actin*, and *RP49* (Table 2).

**Table 2:** A comprehensive ranking of the candidate reference genes using different algorithms*.

*RefFinder*		*geNorm*		*NormFider*		*ΔC*_*t*_		*BestKeeper*			
Ranking	GM	Ranking	SV	Ranking	SV	Ranking	SV	Ranking	r	Ranking	SD
*PRL13*	1.41	*Tubulin*	0.305	*PRL13*	0.134	*PRL13*	0.76	*PRL13*	0.927	*PRL13*	0.49
*v-ATPase*	2.21	*EF1 A*	0.305	*v-ATPase*	0.334	*v-ATPase*	0.79	*18S*	0.888	*v-ATPase*	0.58
*Tubulin*	3.44	*v-ATPase*	0.396	*18S*	0.418	*18S*	0.84	*v-ATPase*	0.860	*GAPDH*	0.62
*18S*	3.66	*PRL13*	0.538	*GAPDH*	0.482	*Tubulin*	0.84	*Tubulin*	0.846	*18S*	0.68
*GAPDH*	4.36	*18S*	0.601	*Tubulin*	0.505	*GAPDH*	0.86	*GAPDH*	0.834	*28S*	0.71
*EF1 A*	4.45	*GAPDH*	0.649	*28S*	0.621	*28S*	0.94	*28S*	0.816	*SDHA*	0.72
*28S*	5.96	*28S*	0.698	*EF1 A*	0.727	*EF1 A*	0.97	*EF1 A*	0.800	*Tubulin*	0.75
*SDHA*	7.44	*SDHA*	0.778	*SDHA*	0.931	*SDHA*	1.15	*RP49*	0.538	*EF1 A*	0.96
*Actin*	9.00	*Actin*	0.874	*Actin*	1.141	*Actin*	1.38	*SDHA*	0.502	*Actin*	1.02
*RP49*	10.00	*RP49*	0.988	*RP49*	1.348	*RP49*	1.44	*Actin*	0.498	*RP49*	1.09

*: Parameters used in this ranking are Geometric Mean (GM), Stability Value (SV), Pearson’s correlation coefficient (r), and Standard Deviation (SD).

In *NormFinder*, a low stability value indicates a more stable gene. Based on the stability value, *RPL13* (0.134) was the most stable and reliable reference gene. The overall ranking of candidate reference genes from the most stable to the least stable was: *RPL13*, *v-ATPase*, *18S*, *GAPDH*, *Tubulin*, *28S*, *EF1A*, *SDHA*, *Actin*, and *RP49* (Table 2).

*BestKeeper* provides a two-way ranking which separates the correlation of expression among the genes from the overall variations in expression levels (SD). Based on *BestKeeper*, *RPL13* (SD = 0.49) and *v-ATPase* (SD = 0.58) had the most stable expression across all the life stages (S3 Table).

### Comprehensive ranking by *RefFinder*

*RefFinder* is a web-based platform integrating all four above-mentioned algorithms to evaluate and select reference genes from extensive experimental datasets. In this study, all algorithms except *geNorm* recommended *RPL13* as the most stable reference gene (Table 2), which is consistent with the ranking from *RefFinder*. The comprehensive ranking of candidate reference genes from the most stable to the least stable was: *RPL13*, *GAPDH*, *v-ATPase*, *18S*, *Tubulin*, *28S*, *EF1A*, *SDHA*, *Actin*, and *RP49*. Among them, GM values of *Actin* and *RP49* were both higher than 9.0 (Table 2), indicating the lowest stability and the least likelihood of serving as the reference gene for qRT-PCR analysis in *T*. *urticae* across different developmental stages.

### Validation of the recommended reference genes

**Fig 3:**
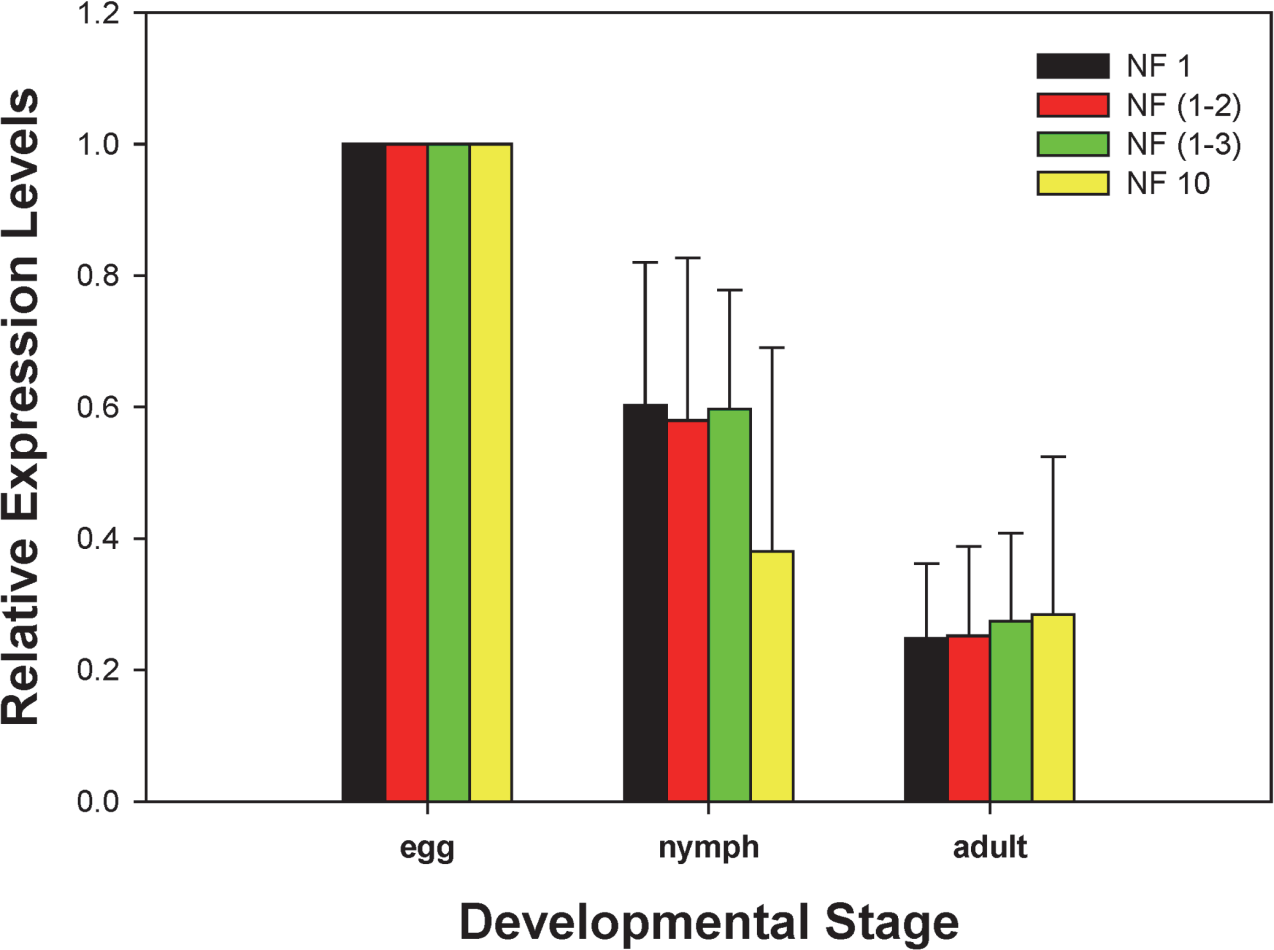
Validation of the recommended reference genes. Expression profiles of *CYP392D8* were investigated using different normalization factors. The expression of *CYP392D8* was normalized using the best reference gene (NF1), the top two [NF (1–2)], the top three reference genes [NF (1–3)], and the worst reference gene (NF10). Bars represent the means and standard error of three biological replications.

To examine the validity of the selected reference genes (genes with the low *Geomean* value are considered stable), the expression profile of a target gene, *CYP392D8*, was investigated under different developmental stages. Similar expression profiles of *CYP392D8* were observed using either one [*RPL13*; NF1], two [*RPL13* and *GAPDH*; NF (1–2)], or three best reference genes [*RPL13*, *GAPDH*, and *v-ATPase*; NF (1–3)] for the normalization (Fig. 3). The expression of *CYP392D8* was numerically lower at the nymph stage using a single reference gene with the highest *Geomean* value (*RP49*; NF10) (Fig. 3).

## Discussion

qRT-PCR quantification demands a comprehensive normalization by reference genes to offset confounding variations among the extensive experimental datasets. Most gene expression studies in the literature use a single endogenous control; this will profoundly influence the statistical outcome and may lead to inaccurate data interpretation [16]. Most recently, there are a influx of reference gene selection studies in insects, including whitefly, diamondback moth, brown planthopper, beet armyworm, oriental leafworm moth, Colorado potato beetle, oriental fruit fly, Russian wheat aphid, and pea aphid, etc [17–25]. In comparison, reference gene selections have largely been ignored in Tetranychidae species. Attribute to their economical importance, carmine spider mite, *T*. *cinnabarinus* (Boisduval) [26], *T*. *urticae* [27], and citrus red mite, *Panonychus citri* (McGregor) [28] are the only three examples in Tetranychidae. In Yue et al. 2013 [27], the mRNA expression profiles of eight housekeeping genes were investigated in fenpropathrin-susceptible and -resistant strains of *T*. *urticae*. Our major concern is that there was not sufficient replication in the experimental design of Yue et al. 2013 [27]; the entire dataset was generated from approximately 400 fenpropathrin-susceptible and 400 fenpropathrin-resistant adult females, respectively. Additionally, our experiments have contrasting designs in that Yue et al. 2013 focused on the selection of reference genes under the impact of insecticide resistance [27], while we investigated the expression profiles of ten housekeeping genes across different developmental stages of *T*. *urticae*.

In our study, except for *18S* and *28S*, the *C*_*t*_ values of these selected reference genes are rather high (S1 Table); however, the *C*_*t*_ values for reference genes are mostly around 15–25 in most of the insects [17–23]. As for the high *C*_*t*_ values, only 0.1 μg of total RNA was used for the cDNA synthesized in this study, whereas, 1.0 μg of total RNA with M-MLV reverse transcription kit was used in previous studies [17–23]. This could be the main factor contributing to the high *C*_*t*_ values. In addition, the expression of reference genes differs in different organisms. For example, the *Actin* gene was highly expressed in the citrus red mite, *Panonychus citri* with the *C*_*t*_ values around 23 across various experimental conditions [28], while, the *Actin* gene was less expressed in the *T*. *urticae* strain in our study with the *C*_*t*_ value around 28. The *Actin* gene was also less expressed in another *T*. *urticae* strain with the *C*_*t*_ value around 27 (Sunny Yoon, University of Kentucky, personal communication), although different primer pairs of the same gene were used between our studies.

Although housekeeping genes are constitutively expressed to maintain cellular function, they do not necessarily meet the prerequisites for a good reference gene that can be ‘expressed at constant levels across various conditions [16–18]. In fact, there are no "universal" reference genes that are stably expressed and suitable for the entire cell and tissue, and various experimental conditions [17–23]. Therefore, customized reference gene selection under specific experimental conditions is highly recommended [29].

There has been ongoing discussion about the optimal number of reference genes required for qRT-PCR analysis. To avoid biased normalization, multiple reference genes have been adopted to analyze gene expression under various experimental conditions. A single reference gene is usually insufficient to normalize the expression of target genes [30]. Results from our validation study with a targeted P450 gene are consistent with the multi-gene normalizer concept, suggesting that the use of two reference genes is sufficient to normalize the expression data and provides more conservative estimation of target gene expression in *T*. *urticae* (Figs. 2B and 3). As a result, we recommend that two internal references are necessary for investigating gene expressions in *T*. *urticae* across different developmental stages. The combined results not only provide a standardized procedure for quantification of gene expression in *T*. *urticae*, but also lay a solid foundation for the genomics and functional genomics research in this emerging pest species.

## Supporting Information

S1 Fig The agrose gel profile of the ten candidate reference genes. M, EZ Load 100 bp Molecular Ruler; Templates in the PCR reactions were as follows: 1) *EF1A;* 2) *SDHA;* 3) *GAPDH;* 4) *Tubulin;* 5) *RPL13;* 6) *RP49;* 7) *18S;* 8) *28S;* 9) *v-ATPase;* and 10) *Actin*.
(TIFF)

S2 Fig Melting curve of the ten candidate reference genes. (TIF)

S1 Table The mean and standard deviation (SD) of the *C*_t_ values of the ten candidate reference gene. (DOCX)

S2 Table Pairwise comparison of candidate reference genes. (DOCX)

S3 Table Ranking of the candidate reference genes based on their crossing point (CP) values by BestKeeper. (DOCX)
