# Supplementary figures and images for: Stably Expressed Housekeeping Genes across Developmental Stages in the Two-Spotted Spider Mite, Tetranychus urticae
Source: PLoS One. 2015 Mar 30;10(3):e0120833. doi: 10.1371/journal.pone.0120833 (PMC4379063; doi:10.1371/journal.pone.0120833)

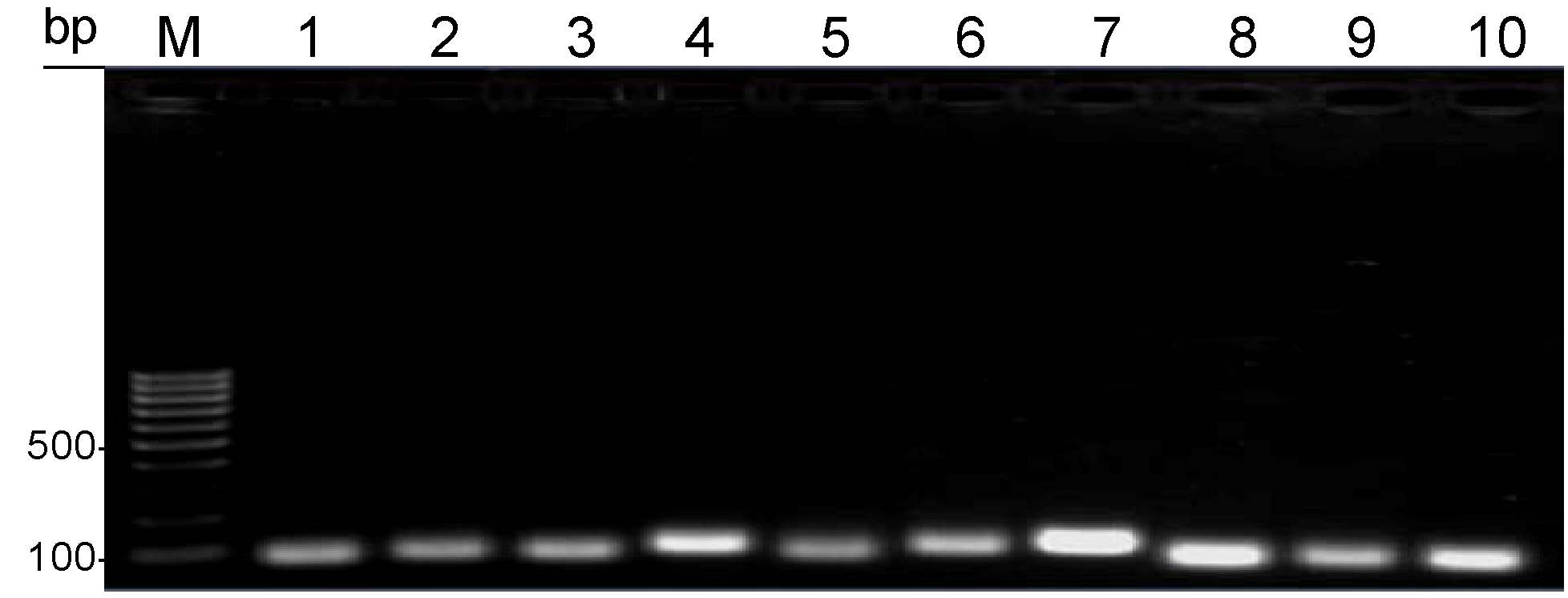

Supplement: S1 Fig — M, EZ Load 100 bp Molecular Ruler; Templates in the PCR reactions were as follows: 1) EF1A; 2) SDHA; 3) GAPDH; 4) Tubulin; 5) RPL13; 6) RP49; 7) 18S; 8) 28S; 9) v-ATPase; and 10) Actin. (TIFF) [file pone.0120833.s001.tiff]

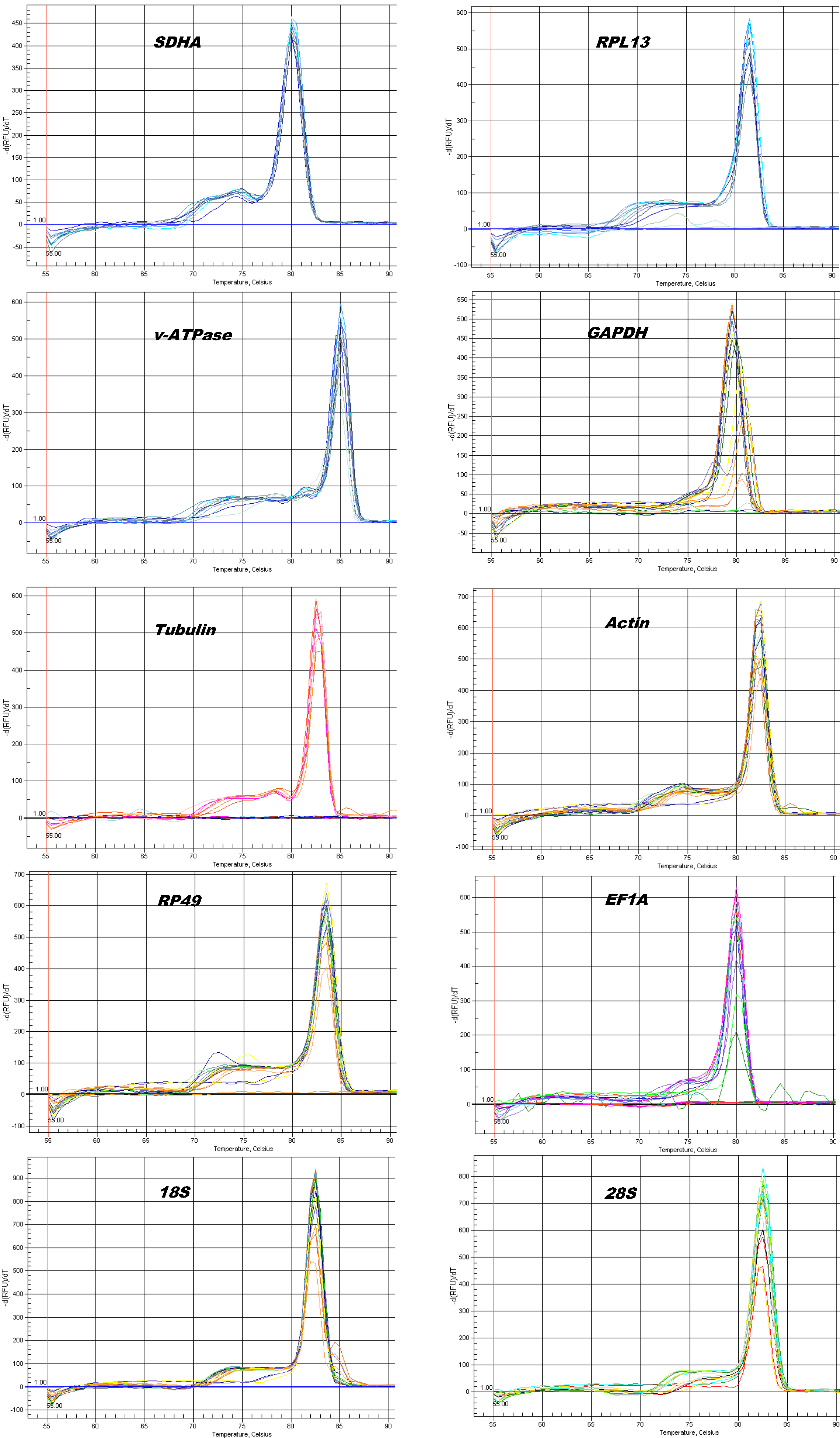

Supplement: S2 Fig — (TIF) [file pone.0120833.s002.tif]
