# Supplementary material for: Stably Expressed Housekeeping Genes across Developmental Stages in the Two-Spotted Spider Mite, Tetranychus urticae
Source: PLoS One. 2015 Mar 30;10(3):e0120833. doi: 10.1371/journal.pone.0120833 (PMC4379063; doi:10.1371/journal.pone.0120833)
Supplement: S1 Table — (DOCX) [file pone.0120833.s003.docx]

**S1 Table. The mean and standard deviation (SD) of the *C_t_* values of the ten candidate reference gene.**

| **Gene** | ***C_t_*** ± **SD** |
| --- | --- |
| *SDHA* | 31.39± 1.02 |
| *RPL13* | 30.39± 0.67 |
| *v-ATPase* | 29.84 ±0.84 |
| *GAPDH* | 29.39± 0.84 |
| *Tubulin* | 28.97± 1.00 |
| *Actin* | 28.53 ±1.23 |
| *RP49* | 28.22 ±1.42 |
| *EF1 A* | 27.22± 1.17 |
| *18S* | 15.89 ±0.84 |
| *28S* | 14.94± 0.94 |
