# Supplementary material for: Stably Expressed Housekeeping Genes across Developmental Stages in the Two-Spotted Spider Mite, Tetranychus urticae
Source: PLoS One. 2015 Mar 30;10(3):e0120833. doi: 10.1371/journal.pone.0120833 (PMC4379063; doi:10.1371/journal.pone.0120833)
Supplement: S2 Table — (DOCX) [file pone.0120833.s004.docx]

**S2 Table. Pairwise comparison of candidate reference genes***

| ***Gene*** |  | **Pair1** | **Pair2** | **Pair3 3 333 3** | **Pair4 4** | **Pair5 55** | **Pair6 6** | **Pair7 7** | **Pair8 8** | **Pair9 9** | **Avg SD** |
| --- | --- | --- | --- | --- | --- | --- | --- | --- | --- | --- | --- |
| *28S* | Mean | 0.95 | 13.59 | 13.28 | 15.44 | 14.03 | 12.27 | 14.89 | 16.45 | 14.45 |  |
|  | SD | 0.56 | 1.46 | 0.89 | 0.64 | 1.02 | 1.22 | 0.95 | 1.19 | 0.53 | 0.94 |
| *18S* | Mean | -0.95 | 12.64 | 12.33 | 14.50 | 13.08 | 11.33 | 13.95 | 15.50 | 13.50 |  |
|  | SD | 0.56 | 1.15 | 1.26 | 0.51 | 0.70 | 0.91 | 0.67 | 1.22 | 0.57 | 0.84 |
| *Actin* | Mean | -13.59 | -12.64 | -0.31 | 1.86 | 0.44 | -1.31 | 1.31 | 2.86 | 0.86 |  |
|  | SD | 1.56 | 1.23 | 2.11 | 1.30 | 1.05 | 1.06 | 0.98 | 1.57 | 1.54 | 1.38 |
| *RP49* | Mean | -13.28 | -12.33 | 0.31 | 2.16 | 0.75 | -1.01 | 1.61 | 3.17 | 1.17 |  |
|  | SD | 0.89 | 1.26 | 2.01 | 1.14 | 1.71 | 1.86 | 1.55 | 1.73 | 0.86 | 1.45 |
| *RPL13* | Mean | -15.44 | -14.50 | -1.86 | -2.16 | -1.42 | -3.17 | -0.55 | 1.00 | -0.99 |  |
|  | SD | 0.64 | 0.51 | 1.22 | 1.14 | 0.68 | 0.82 | 0.54 | 0.92 | 0.35 | 0.76 |
| *Tubulin* | Mean | -14.03 | -13.08 | -0.44 | -0.75 | 1.42 | -1.75 | 0.87 | 2.42 | 0.42 |  |
|  | SD | 1.02 | 0.70 | 1.00 | 1.71 | 0.68 | 0.30 | 0.36 | 0.88 | 0.92 | 0.84 |
| *EF1A* | Mean | -12.27 | -11.33 | 1.31 | 1.01 | 3.17 | 1.75 | 2.62 | 4.17 | 2.17 |  |
|  | SD | 1.22 | 0.91 | 1.02 | 1.86 | 0.82 | 0.30 | 0.53 | 0.96 | 1.07 | 0.97 |
| *v-ATPase* | Mean | -14.89 | -13.95 | -1.31 | -1.61 | 0.55 | -0.87 | -2.62 | 1.55 | -0.45 |  |
|  | SD | 0.95 | 0.67 | 0.92 | 1.55 | 0.54 | 0.36 | 0.53 | 0.81 | 0.81 | 0.79 |
| *SDHA* | Mean | -16.45 | -15.50 | -2.86 | -3.17 | -1.00 | -2.42 | -4.17 | -1.55 | -2.00 |  |
|  | SD | 1.19 | 1.22 | 1.48 | 1.73 | 0.92 | 0.88 | 0.96 | 0.81 | 1.16 | 1.15 |
| *GAPDH* | Mean | -14.45 | -13.50 | -0.86 | -1.17 | 0.99 | -0.42 | -2.17 | 0.45 | 2.00 |  |
|  | SD | 0.53 | 0.57 | 1.45 | 0.86 | 0.35 | 0.92 | 1.07 | 0.81 | 1.16 | 0.86 |

"*": *ΔCt* method was used for the data analysis.
