# Supplementary material for: Stably Expressed Housekeeping Genes across Developmental Stages in the Two-Spotted Spider Mite, Tetranychus urticae
Source: PLoS One. 2015 Mar 30;10(3):e0120833. doi: 10.1371/journal.pone.0120833 (PMC4379063; doi:10.1371/journal.pone.0120833)
Supplement: S3 Table — (DOCX) [file pone.0120833.s005.docx]

**S3 Table. Ranking of the candidate reference genes based on their crossing point (CP) values by *BestKeeper****

| **Gene** | **GM** | **AM** | **Min** | **Max** | **SD** | **CV** | **[r]** | ***p*-value** | **Ranking** | |
| --- | --- | --- | --- | --- | --- | --- | --- | --- | --- | --- |
|  |  |  |  |  |  |  |  |  | **[r]** | **SD** |
| *28S* | 14.91 | 14.94 | 13.06 | 15.94 | 0.71 | 4.77 | 0.816 | 0.007 | *PRL13* | *PRL13* |
| *18S* | 15.87 | 15.89 | 14.47 | 16.84 | 0.68 | 4.29 | 0.888 | 0.001 | *18S* | *v-ATPase* |
| *Actin* | 28.50 | 28.53 | 26.86 | 30.5 | 1.02 | 3.57 | 0.498 | 0.172 | *v-ATPase* | *GAPDH* |
| *RP49* | 28.19 | 28.22 | 26.45 | 30.89 | 1.09 | 3.88 | 0.538 | 0.136 | *Tubulin* | *18S* |
| *PRL13* | 30.38 | 30.39 | 29.05 | 31.07 | 0.49 | 1.61 | 0.927 | 0.001 | *GAPDH* | *28S* |
| *Tubulin* | 28.95 | 28.97 | 26.99 | 30.17 | 0.75 | 2.59 | 0.846 | 0.004 | *28S* | *SDHA* |
| *EF1 A* | 27.19 | 27.22 | 25.07 | 28.47 | 0.96 | 3.54 | 0.800 | 0.010 | *EF1 A* | *Tubulin* |
| *v-ATPase* | 29.83 | 29.84 | 28.07 | 31.12 | 0.58 | 1.95 | 0.860 | 0.003 | *RP49* | *EF1 A* |
| *SDHA* | 31.37 | 31.39 | 29.21 | 32.66 | 0.72 | 2.29 | 0.502 | 0.169 | *SDHA* | *Actin* |
| *GAPDH* | 29.38 | 29.39 | 28.00 | 30.49 | 0.62 | 2.09 | 0.834 | 0.005 | *Actin* | *RP49* |

"*": Two criteria are considered: Pearson’s correlation coefficient and *BestKeeper* computed SD values. The stability of a gene is directly proportional to the [r] value, while it is inversely proportional to the SD value.

GM: the geometric mean of CP; AM: the arithmetic mean of CP; Min and Max: the extreme values of CP; SD: the standard deviation of the CP; CV: the coefﬁcient of variance expressed as a percentage on the CP level; [r]: Pearson’s correlation coefficient.
